# Phenome-wide causal associations between osteoarthritis and other complex traits through the latent causal variable analysis

**DOI:** 10.1186/s12891-024-07360-x

**Published:** 2024-03-26

**Authors:** Lin Mei, Zhiming Zhang, Ruiqi Chen, Zhihong Li

**Affiliations:** 1 Department of Orthopedics, The Second Xiangya Hospital, Central South University, Changsha, China; 2 Hunan Key Laboratory of Tumor Models and Individualized Medicine, The Second Xiangya Hospital, Changsha, China

**Keywords:** Osteoarthritis, Causal inference, Genome-wide association study, Genetic correlation, Latent causal variable study

## Abstract

**Background:**

Individuals with osteoarthritis present with comorbidities, and the potential causal associations remain incompletely elucidated. The present study undertook a large-scale investigation about the causality between osteoarthritis and variable traits, using the summary-level data of genome-wide association studies (GWAS).

**Methods:**

The present study included the summary-level GWS data of knee osteoarthritis, hip osteoarthritis, hip or knee osteoarthritis, hand osteoarthritis, and other 1355 traits. Genetic correlation analysis was conducted between osteoarthritis and other traits through cross-trait bivariate linkage disequilibrium score regression. Subsequently, latent causal variable analysis was performed to explore the causal association when there was a significant genetic correlation. Genetic correlation and latent causal variable analysis were conducted on the Complex Traits Genomics Virtual Lab platform (https://vl.genoma.io/).

**Results:**

We found 133 unique phenotypes showing causal relationships with osteoarthritis. Our results confirmed several well-established risk factors of osteoarthritis, such as obesity, weight, BMI, and meniscus derangement. Additionally, our findings suggested putative causal links between osteoarthritis and multiple factors. Socioeconomic determinants such as occupational exposure to dust and diesel exhaust, extended work hours exceeding 40 per week, and unemployment status were implicated. Furthermore, our analysis revealed causal associations with cardiovascular and metabolic disorders, including heart failure, deep venous thrombosis, type 2 diabetes mellitus, and elevated cholesterol levels. Soft tissue and musculoskeletal disorders, such as hallux valgus, internal derangement of the knee, and spondylitis, were also identified to be causally related to osteoarthritis. The study also identified the putative causal associations of osteoarthritis with digestive and respiratory diseases, such as Barrett’s esophagus, esophagitis, and asthma, as well as psychiatric conditions including panic attacks and manic or hyperactive episodes. Additionally, we observed osteoarthritis causally related to pharmacological treatments, such as the use of antihypertensive medications, anti-asthmatic drugs, and antidepressants.

**Conclusion:**

Our study uncovered a wide range of traits causally associated with osteoarthritis. Further studies are needed to validate and illustrate the detailed mechanism of those causal associations.

**Supplementary Information:**

The online version contains supplementary material available at 10.1186/s12891-024-07360-x.

## Introduction

Osteoarthritis (OA) is a degenerative joint disease characterized by cartilage degradation, subchondral osteosclerosis, osteophyte development, and diminution of the joint space, causing joint discomfort and rigidity. Data from 2020 have indicated that OA has a global prevalence, affecting approximately 595 million individuals, marking an escalation of 132.2% from 1990, and constituting 7.6% of the worldwide populace. Owing to population expansion and increased life expectancy, previous study has suggested a 74.9% increase in knee OA cases and a 78.6% increase in hip OA cases by 2050 [1].

Several risk factors have been delineated in correlation with OA onset and progression, including age, gender disparities, socioeconomic status, family predisposition, obesity, high blood pressure, and augmented bone mineral density [2]. Previous studies have reported the comorbidities in OA, such as subclinical atherosclerosis, cardiovascular diseases, metabolic syndrome, and psychological conditions like depression and anxiety [3–6]. Recently, Kamps and colleagues have cataloged 42 comorbidities in individuals with OA, including cardiovascular, metabolic, musculoskeletal, neurological, psychiatric, gastrointestinal, liver, urological, internal, dermatological, and other related conditions [7].

Beyond the traditional epidemiologic studies, genetic studies have yielded novel insights into the relationships between OA and other complex traits. While the previous study has discerned significant genetic correlations between OA and 35 out of 219 phenotypes [8], the genetic correlations between OA and other traits may be explained by horizontal pleiotropy or by vertical pleiotropy. The horizontal pleiotropy, which means the genetic variants directly affect both traits, not via each other, complicates the elucidation of causal relationships between OA and diverse traits. Mendelian randomization (MR) studies have been also conducted to investigate the causal relationships between OA and other traits, such as anthropometrics, cardiovascular disorders, socioeconomic conditions, and among others [9–13]. However, those studies focused exclusively on a limited number of traits.

The present study has aimed to conduct a large-scale investigation about the causal relationships between OA and different traits, using genome-wide association studies (GWAS) summary statistics of OA and more than 1300 phenotypes.

## Materials and methods

### GWAS summary-level datasets

The present study included four OA subtypes, including knee OA (KOA), hip OA (HOA), hip or knee OA (HKOA), and hand OA. We used the summary-level GWAS data for KOA (ID: ebi-a-GCST007090), HOA (ebi-a-GCST007091), and HKOA (ebi-a-GCST007092) from Tachmazidou’s study [14], which were downloaded from OpenGWAS (https://gwas.mrcieu.ac.uk/datasets/). The GWAS data for hand OA were retrieved from Boer’s study [15]. Sample sizes of the GWAS summary statistics varied from 303,782 to 417,596 individuals of European ancestry. The comprehensive analytical methodology of the GWAS study was demonstrated in the published study [14, 15].

For our analysis, the following parameters were extracted from the OA GWAS summary statistics: rsID, chromosomal coordinates, effect allele, other allele, allele frequency, effect size, standard error, and the p-value of each genetic variant. Variants from either chromosome X or chromosome Y were filtered out.

### Complex traits Genomics virtual lab

The Complex Traits Genomics Virtual Lab (CTG-VL) (https://vl.genoma.io/) served as a web-based platform, hosting more than 1600 GWAS summary statistics primarily derived from European ancestry [16, 17]. Predominantly, these post-GWAS data were sourced from the Neale Lab, as documented on their official repository (http://www.nealelab.is/uk-biobank/). In the present study, to explore the relationships with OA, we leveraged the GWAS summary statistics from European ancestry for 1355 phenotypes available in the CTG-VL platform to conduct the latent causal variable (LCV) analysis. This analytical approach was facilitated by the Phenome-wide LCV pipeline (https://github.com/lukejoconnor/LCV), an integral component of the CTG-VL platform [18, 19]. A comprehensive elucidation of the Phenome-wide LCV pipeline’s methodology was available in the previous publication [20].

In this study, we uploaded the summary-level GWAS data on the CTG-VL platform and selected the LCV analysis function to conduct the analysis.

### Genetic correlation and latent causal variable analysis

The genetic correlations between OA and various traits were quantified utilizing linkage disequilibrium score regression [21]. Upon discerning a significant genetic correlation between OA and a given trait (denoted as trait X), it became feasible to compute the genetic causality proportion (GCP) via the LCV approach [20]. This computation operated under the premise of a latent variable, denoted as L, which served as a mediator for the observed genetic correlation. The potential values for GCP spanned from − 1 to 1. A GCP value of 0 signified that horizontal pleiotropy underpinned the genetic correlation between OA and trait X. Conversely, a GCP value of 1 implied a complete genetic causality of OA for trait X. A GCP value within the range of 0 < |GCP| < 1 indicated a partial genetic causality between OA and trait X.

### Statistical analysis

For multiple testing correction, we employed the Benjamini–Hochberg False Discovery Rate (FDR) < 0.05 as a threshold for both genetic correlations and GCP estimates. It’s noteworthy that the capacity to discern a genetic causal association diminished for GCP values ranging between − 0.6 and 0.6 [20]. Therefore, |GCP| > 0.6 was set as another threshold for the LCV analysis.

## Results

**Fig. 1:**
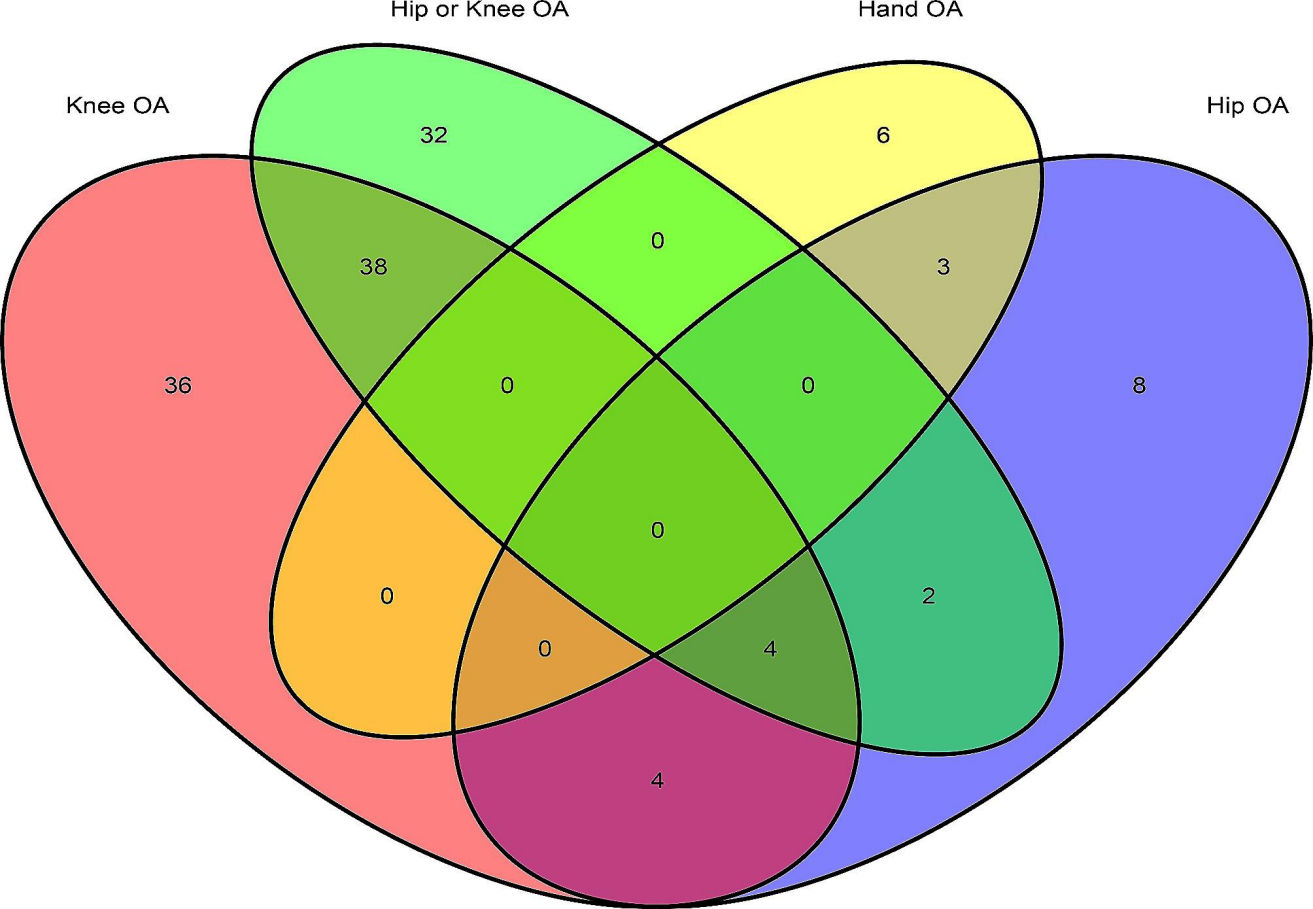
The Venn diagram for the traits with causal relationships with knee OA, hip OA, hip or knee OA, and hand OA

The present studies included 1355 phenotypes to investigate their relationships with OA (Additional file S1). The causal associations were identified between OA and 133 distinct phenotypes (Fig. 1). Of these, four associations were common across KOA, HOA, and KHOA, including BMI, meniscus derangement, weight, and gained weight during worst episode of depression. Furthermore, an additional three associations were identified as shared between HOA and hand OA, such as hip pain experienced in last month, leg pain on walking, and glucosamine supplements.

### Knee OA

**Fig. 2:**
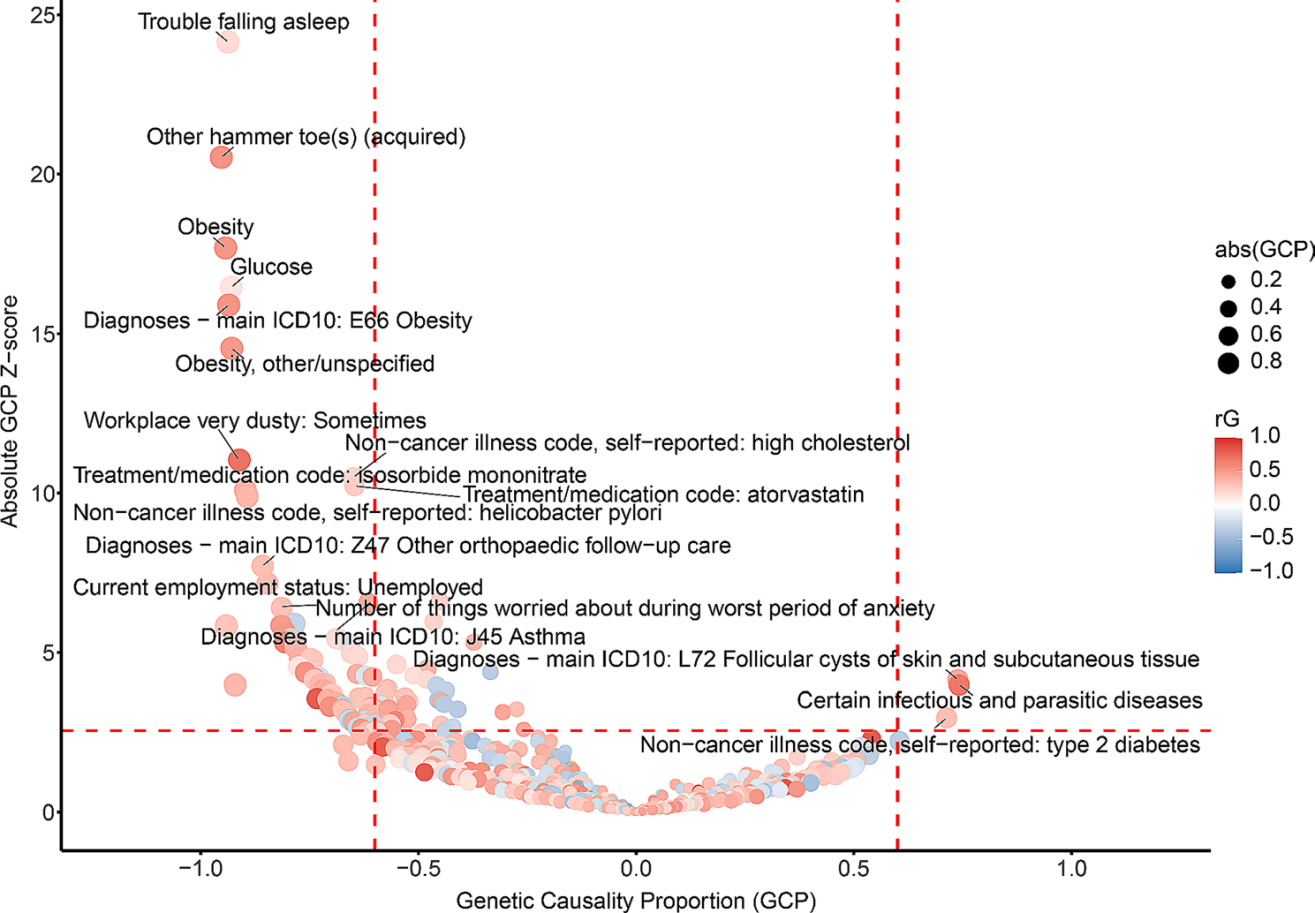
The causal relationships between knee OA and diverse traits. Each dot represented one trait with significant genetic correlation with knee OA (FDR < 0.05). Traits in red showed positive genetic correlations with knee OA, while those in blue demonstrated negative genetic correlations. Both FDR < 0.05 and |GCP| > 0.6 were set as the significant threshold (the red dashed lines) for GCP estimates. Traits with GCP < -0.6 could causally affect knee OA, while traits could be causally influenced by knee OA if GCP > 0.6

We identified 592 traits that showed significant genetic correlations with KOA (FDR < 0.05) (Additional file S2). A total of 82 phenotypes showed inferred causal associations with KOA (FDR < 0.05, |GCP| > 0.6) (Fig. 2; Table 1; Additional file S3).

**Table 1:** Phenotypes with causal relationships with knee OA

Phenotype	rG	GCP	P_GCP
Certain infectious and parasitic diseases	0.678	0.742	6.74E-05
Diagnoses - main ICD10: L72 Follicular cysts of skin and subcutaneous tissue	0.434	0.739	3.47E-05
Non-cancer illness code, self-reported: type 2 diabetes	0.320	0.713	3.11E-03
Hip circumference	0.408	-0.606	2.31E-05
Diagnoses - main ICD10: J45 Asthma	0.429	-0.615	5.56E-11
Weight	0.397	-0.641	1.52E-05
Treatment/medication code: atorvastatin	0.252	-0.647	8.07E-25
Non-cancer illness code, self-reported: high cholesterol	0.207	-0.649	4.46E-26
Diagnoses - main ICD10: I50 Heart failure	0.301	-0.672	1.47E-03
Heel bone mineral density (BMD) (left)	0.190	-0.684	6.38E-08
Work hours - lumped category: Over 40 h	0.278	-0.701	2.26E-04
BMI	0.400	-0.708	4.03E-04
Chest pain or discomfort when walking uphill or hurrying	0.364	-0.727	6.02E-05
Meniscus derangement	0.801	-0.733	3.74E-04
IPF (EUR Biobanks)	0.333	-0.782	2.72E-07
Job SOC coding: Higher education teaching professionals	-0.315	-0.799	8.43E-08
Current employment status: Unemployed	0.342	-0.847	7.97E-13
Workplace very dusty: Sometimes	0.702	-0.911	1.19E-28
Glucose	0.138	-0.930	3.55E-61
Treatment/medication code: beclometasone	0.296	-0.941	5.50E-09
Obesity	0.526	-0.943	2.22E-70

Phenotypes with FDR < 0.05 and |GCP| > 0.6. The whole list of phenotypes with significant causal associations with knee OA was provided in Additional file S3, where detailed information of those was provided. *OA* osteoarthritis, *rG* genetic correlation, *GCP* genetic causality proportion, *SE* standard error, *P p* value

Among those, KOA was identified to increase the risks for type 2 diabetes (T2D), certain infectious and parasitic diseases, and follicular cysts of skin and subcutaneous tissue (GCP > 0.6, FDR < 0.05).

For the remaining 79 traits that exhibited putative causal effects on KOA (GCP < -0.6, FDR < 0.05), several risk factors observed in epidemiologic studies were identified to augment the susceptibility to KOA in this study, such as meniscus derangement, obesity, weight, hip circumference, bone mineral density, and high cholesterol.

We ascertained that certain socioeconomic status might potentially increase the risk of KOA, including occupational exposures such as dusty environments, elevated temperatures, and pronounced diesel exhaust presence, in addition to unemployment and extended working hours > 40 h. Conversely, specific employment statuses and roles, including salaried employment or self-employed, higher education teaching professionals, and medical practitioners, might be inversely associated with KOA susceptibility.

Notably, several cardiovascular and metabolic conditions were found as the potential amplifiers of KOA risk, inclusive of heart failure, high cholesterol, and glucose. Our findings suggested that KOA might be a plausible causal consequence of gastrointestinal diseases (such as Barrett’s esophagus, esophagitis, ventral hernia, intestinal malabsorption, and Helicobacter pylori infection) and pulmonary disorders (like asthma and idiopathic pulmonary fibrosis). Intriguingly, anti-asthmatic medication, beclomethasone in particular, exhibited a positive correlation with KOA.

Pharmacological interventions displayed positive correlations with KOA, encompassing anti-hypertensive agents (perindopril, isosorbide mononitrate, doxazosin, and bisoprolol), anti-depression treatment (amitriptyline), and statin medication (atorvastatin).

### Hip OA

**Fig. 3:**
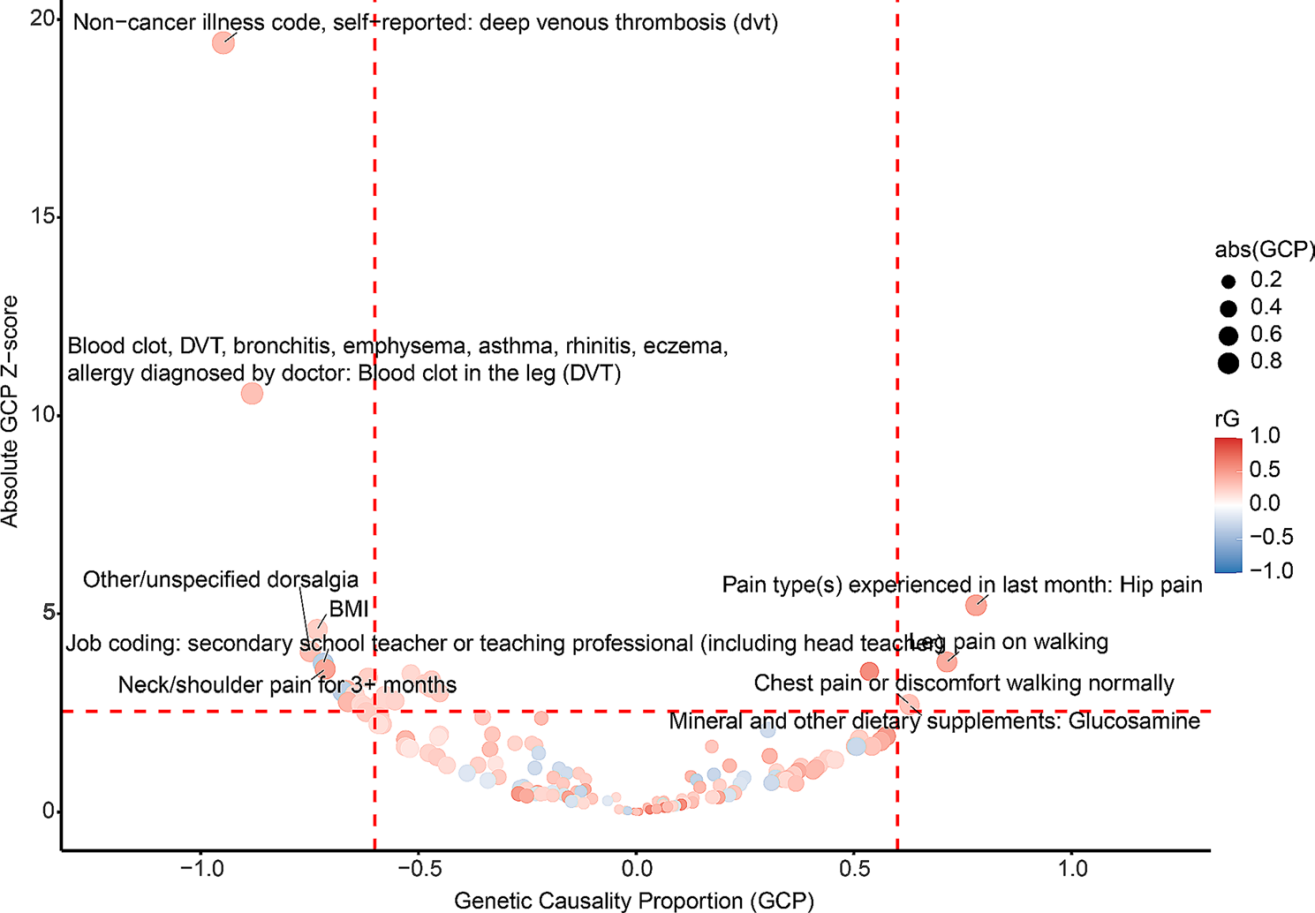
The causal relationships between hip OA and diverse traits. Each dot represented one trait with significant genetic correlation with hip OA (FDR < 0.05). Traits in red showed positive genetic correlations with hip OA, while those in blue demonstrated negative genetic correlations. Both FDR < 0.05 and |GCP| > 0.6 were set as the significant threshold (the red dashed lines) for GCP estimates. Traits with GCP < -0.6 could causally affect hip OA, while traits could be causally influenced by hip OA if GCP > 0.6

There were 165 traits significantly genetically correlated with hip OA after multiple testing correction (FDR < 0.05) (Additional file S4). We found the causal associations between hip OA and 21 phenotypes in total (Fig. 3; Table 2; Additional file S5).

**Table 2:** Phenotypes with causal relationships with hip OA

Phenotype	rG	GCP	P_GCP
Pain type(s) experienced in last month: Hip pain	0.443	0.781	1.86E-07
Leg pain on walking	0.420	0.714	1.56E-04
Mineral and other dietary supplements: Glucosamine	0.297	0.628	6.88E-03
Chest pain or discomfort walking normally	0.251	0.627	6.27E-03
Weight	0.239	-0.616	7.05E-04
Arm predicted mass (right)	0.202	-0.625	7.66E-03
Whole body fat-free mass	0.182	-0.627	7.37E-03
Whole body water mass	0.183	-0.628	7.25E-03
Arm fat-free mass (right)	0.204	-0.631	6.55E-03
Arm fat-free mass (left)	0.207	-0.643	4.46E-03
Arm predicted mass (left)	0.203	-0.644	4.37E-03
Basal metabolic rate	0.201	-0.646	3.15E-03
Meniscus derangement	0.358	-0.662	5.42E-03
Weight change during worst episode of depression: Gained weight	0.346	-0.666	2.15E-03
Job SOC coding: Secondary education teaching professionals	-0.330	-0.674	2.34E-03
Neck/shoulder pain for 3 + months	0.451	-0.714	3.17E-04
Job coding: secondary school teacher or teaching professional (including head teacher)	-0.340	-0.718	1.80E-04
BMI	0.239	-0.732	4.18E-06
Other/unspecified dorsalgia	0.323	-0.749	5.21E-05
Blood clot, DVT, bronchitis, emphysema, asthma, rhinitis, eczema, allergy diagnosed by doctor: Blood clot in the leg (DVT)	0.316	-0.882	2.19E-26
Non-cancer illness code, self-reported: deep venous thrombosis (dvt)	0.336	-0.948	3.12E-84

Phenotypes with FDR < 0.05 and |GCP| > 0.6. More detailed information of the phenotypes was provided in Additional file S5. *OA* osteoarthritis, *rG* genetic correlation, *GCP* genetic causality proportion, *SE* standard error, *P p* value

Hip OA was found to causally increase hip pain, leg pain on walking, chest pain or discomfort walking normally, and the utilization of glucosamine supplement.

Similar to knee OA, several anthropometrics showed the putative influence on hip OA, such as BMI, weight, arm predicted mass, arm predicted mass, whole body fat-free mass, and whole body water mass. Hip OA could be the putative causal outcomes of meniscus derangement and deep venous thrombosis. Furthermore, the occupations in education (secondary school teacher or teaching professional, secondary education teaching professionals) were found to be inversely associated with hip OA risk.

### Hip or knee OA

**Fig. 4:**
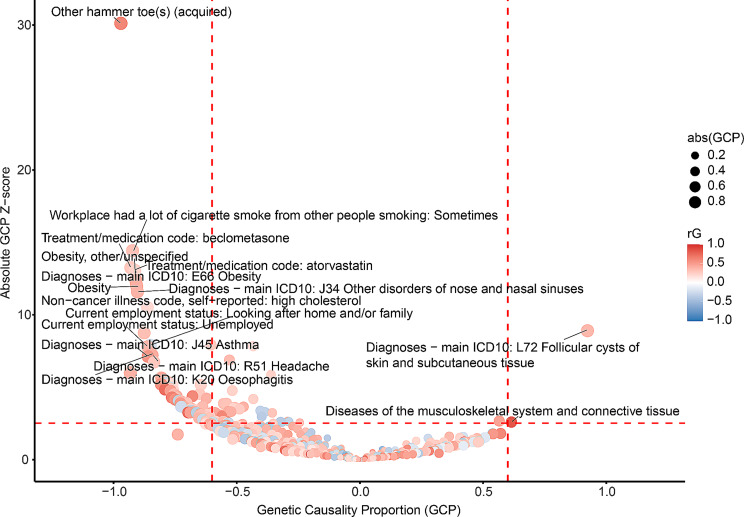
The causal relationships between hip or knee OA and diverse traits. Each dot represented one trait with significant genetic correlation with hip or knee OA (FDR < 0.05). Traits in red showed positive genetic correlations with hip OA, while those in blue demonstrated negative genetic correlations. Both FDR < 0.05 and |GCP| > 0.6 were set as the significant threshold (the red dashed lines) for GCP estimates. Traits with GCP < -0.6 could causally affect hip or knee OA, while traits could be causally influenced by hip or knee OA if GCP > 0.6

Genetic correlations were observed between HKOA and 509 phenotypes in all (Additional file S6). Among those, seventy-six traits could be causally associated with HKOA (Fig. 4; Table 3; Additional file S7).

**Table 3:** Phenotypes with causal relationships with hip or knee OA

Phenotype	rG	GCP	P_GCP
Diagnoses - main ICD10: L72 Follicular cysts of skin and subcutaneous tissue	0.358	0.924	2.66E-19
Diseases of the musculoskeletal system and connective tissue	0.840	0.615	9.33E-03
Work hours - lumped category: Over 40 h	0.298	-0.613	3.72E-03
Workplace had a lot of cigarette smoke from other people smoking: Often	0.255	-0.616	1.67E-03
Weight	0.392	-0.623	2.28E-05
Job SOC coding: Higher education teaching professionals	-0.264	-0.638	5.35E-03
Hallux valgus (acquired)	0.347	-0.639	2.59E-04
Heel bone mineral density (BMD)	0.226	-0.645	2.26E-07
FI10 : arithmetic sequence recognition	-0.342	-0.663	1.18E-03
Workplace very hot: Often	0.386	-0.670	8.46E-04
Workplace very dusty: Sometimes	0.596	-0.679	1.17E-05
Job SOC coding: Secondary education teaching professionals	-0.319	-0.686	7.05E-04
Diagnoses - main ICD10: M23 Internal derangement of knee	0.729	-0.697	1.10E-03
FI8 : chained arithmetic	-0.242	-0.726	2.49E-04
Glycated haemoglobin (HbA1c)	0.167	-0.761	2.85E-07
Non-cancer illness code, self-reported: back problem	0.325	-0.789	7.17E-08
Meniscus derangement	0.730	-0.791	1.19E-06
Current employment status: Unemployed	0.374	-0.862	5.69E-15
Obesity	0.406	-0.906	2.62E-33
Workplace had a lot of cigarette smoke from other people smoking: Sometimes	0.317	-0.923	1.76E-47
Non-cancer illness code, self-reported: chronic obstructive airways disease/copd	0.394	-0.932	2.03E-09

Phenotypes with FDR < 0.05 and |GCP| > 0.6. The whole list of phenotypes with significant causal associations with hip or knee OA was provided in Additional file S7, where detailed information of those was provided. *OA* osteoarthritis, *rG* genetic correlation, *GCP* genetic causality proportion, *SE* standard error, *P* p value

The traits with the evidence of causal outcomes of HKOA were follicular cysts of skin and subcutaneous tissue, and diseases of the musculoskeletal system and connective tissue.

Analogous to the KOA and HOA, the probability of developing HKOA might be reduced among individuals in academic professions, particularly among higher education teaching professionals and secondary education teaching professionals. Furthermore, cognitive and educational factors, such as FI10 (arithmetic sequence recognition) and FI8 (chained arithmetic), were putatively inversely associated with HKOA.

Our study revealed that various socioeconomic determinants were associated with an elevated risk of HKOA. These included unemployed status, working more than 40 h, exposure to significant cigarette smoke in the workplace, and working conditions characterized by extreme heat or excessive dust. These findings aligned with previous results focused on KOA exclusively.

Furthermore, our analysis indicated that biomechanical and metabolic factors, such as elevated BMI, increased weight, higher arm fat mass, increased heel bone mineral density, and obesity, could amplify the risk of HKOA.

From a musculoskeletal perspective, conditions such as hallux valgus (acquired), internal derangement of knee, meniscus derangement, spine arthritis/spondylitis, and poly-arthropathies were found to have potential causative links with HKOA, thereby possibly heightening the risk of its development. Additionally, respiratory disorders, including asthma and idiopathic pulmonary fibrosis, demonstrated a causal association with HKOA. Our study also identified a causal relationship between chronic obstructive pulmonary disease (COPD) and HKOA.

In terms of pharmacological influences, certain medications were identified as being positively associated with the risk of HKOA. These encompassed antidepressants (amitriptyline), antidiabetic agents (pioglitazone), antihypertensive medications (including perindopril, doxazosin, and bisoprolol), analgesics (codeine), and anti-asthmatic drugs (beclomethasone).

### Hand OA

**Fig. 5:**
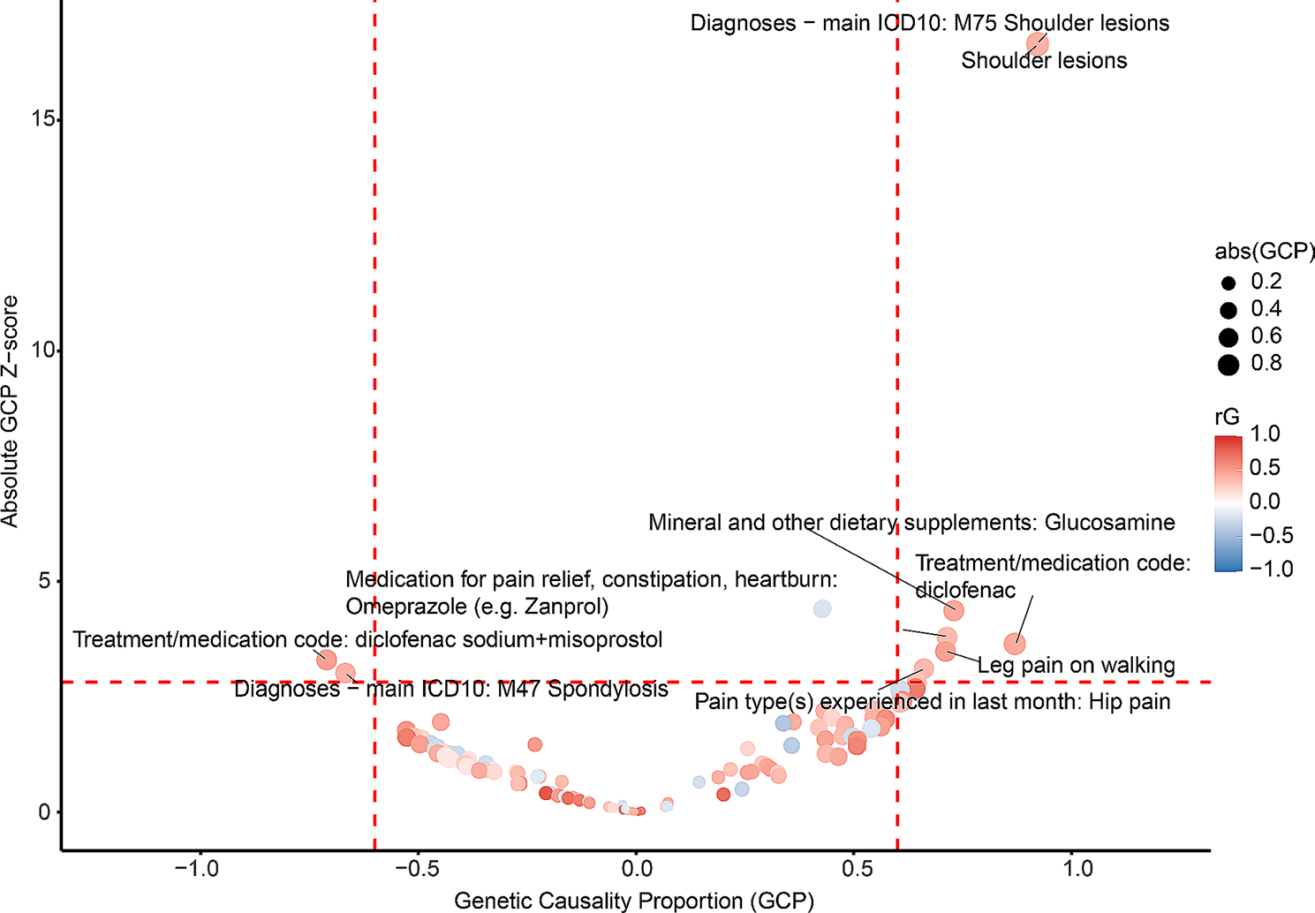
The causal relationships between hand OA and diverse traits. Each dot represented one trait with significant genetic correlation with hand OA (FDR < 0.05). Traits in red showed positive genetic correlations with hand OA, while those in blue demonstrated negative genetic correlations. Both FDR < 0.05 and |GCP| > 0.6 were set as the significant threshold (the red dashed lines) for GCP estimates. Traits with GCP < -0.6 could causally affect hand OA, while traits could be causally influenced by hand OA if GCP > 0.6

We identified 122 traits significantly correlated with hand OA (FDR < 0.05) (Additional file S8), and nine of those showed putative causal associations with hand OA (FDR < 0.05, |GCP| > 0.6) (Fig. 5; Table 4).

**Table 4:** Phenotypes with causal relationships with hand OA

Phenotype	rG	GCP	P_GCP
Treatment/medication code: diclofenac sodium + misoprostol	0.471	-0.711	9.84E-04
Diagnoses - main ICD10: M47 Spondylosis	0.385	-0.668	2.62E-03
Pain type(s) experienced in last month: Hip pain	0.345	0.661	1.90E-03
Leg pain on walking	0.470	0.711	5.03E-04
Medication for pain relief, constipation, heartburn: Omeprazole (e.g. Zanprol)	0.347	0.714	1.54E-04
Mineral and other dietary supplements: Glucosamine	0.435	0.730	1.28E-05
Treatment/medication code: diclofenac	0.428	0.870	2.69E-04
Shoulder lesions	0.393	0.922	1.88E-62
Diagnoses - main ICD10: M75 Shoulder lesions	0.394	0.922	1.11E-62

Phenotypes with FDR < 0.05 and |GCP| > 0.6. *OA* osteoarthritis, *rG* genetic correlation, *GCP* genetic causality proportion, *SE* standard error, *P* p value

Our results demonstrated that hand OA could increase the risk of pain-related traits, including hip pain and leg pain on walking. Hand OA also showed a positive correlation with diclofenac medication, glucosamine supplement, shoulder lesions, and spondylosis.

## Discussion

In this investigation, a comprehensive phenome-wide LCV analysis was executed, revealing causal relationships with KOA, HOA, HKOA, and hand OA for 82, 21, 76, and 9 traits respectively. Consequently, a total of 133 distinct phenotypes were identified. These phenotypes span a diverse array of domains, including anthropometrics, socioeconomic determinants, cardiovascular and metabolic disorders, soft tissue and musculoskeletal disorders, digestive/respiratory diseases, psychiatric conditions, pharmacological interactions, and other miscellaneous conditions.

Previous literature has robustly documented the associations between OA and multiple anthropometric parameters. The causative roles of these factors have been corroborated through MR studies, highlighting variables such as BMI ( [9, 10, 22–25], obesity [26], body weight [9], hip circumference [9, 10], waist circumference [9, 10], and BMD [27–29]. This current analysis further substantiated these causal relationships via the LCV methodology. Additionally, this research elucidated the causative impact of glucose levels, basal metabolic rates, and glycated haemoglobin (HbA1c) on KOA, HOA, and HKOA, respectively, and an elevated risk of T2D was discerned in conjunction with KOA. The causal association of HbA1c was exclusively observed with KOA, but not with HOA in a previous MR study [30]. Moreover, there were inconsistent results about the relationship between T2D and OA, one MR study confirmed that KOA could be the potential risk for T2D [31], whereas another two MR studies failed to establish any causal relationship between T2D and OA [32, 33].

Regarding of the occupational exposures, our LCV analysis revealed that several conditions could increase the risk of developing KOA and HKOA, such as working hours > 40 h, the dusty or hot working environment, cigarette smoke, and the pronounced diesel exhaust presence. Consistently, prior epidemiological studies found that the exposure to fine particulate matter could increase the risk of KOA [34, 35]. Furthermore, cell-based and animal-based experiments showed that particulate matter could aggravated the OA severity via increasing pro-inflammatory factors [36, 37]. In concordance with the result from an observational study suggesting that lower physical work was associated with reduced risk of OA [38], from a genetic perspective, the present study revealed that jobs, including salaried employment or self-employed, higher education teaching professionals, and medical practitioners, might be inversely associated with OA susceptibility, which was increased under the unemployment condition.

In terms of cardiovascular diseases, coronary atherosclerosis was observed to confer a protective effect against OA, while OA itself emerged as a potential risk determinant for atrial fibrillation [11]. Another MR study probed the influence of OA on a suite of 14 cardiovascular diseases, concluding that KOA could elevate the susceptibility to venous thromboembolism and pulmonary embolism and HOA was linked to augmented risks of coronary artery disease, atrial fibrillation, and ischemic stroke. However, it did not detect any causal effect on heart failure from either KOA or HOA [39], and Zhao and colleagues did not affirm the causative influence of KOA or HOA on heart failure [12]. Contrasting these findings, our study found that heart failure showed causal effect on both KOA and HKOA, but not on HOA. Furthermore, the manifestation of chest pain during ambulation was concurrently associated with KOA, HOA, and HKOA.

Data from German Health Update demonstrated that the OA incidence was significantly higher in individuals with asthma compared with those without asthma, and the increased risk remained significant even after adjusted for age group, gender, BMI, educational attainment, and smoking status [40]. The above result was supported by another nationwide survey [41]. However, MR studies found that asthma did not show a causal effect on OA [42, 43]. In the present study, we identified the elevated risk of KOA and HKOA in individuals with asthma through the LCV approach, which showed higher power to detect the causal relationship compared to traditional MR methods, as discussed in the original study [20].

Several limitations were present in the study. First, although we only studied the causal associations with more than 1300 phenotypes, other traits need to be further explored. Second, since we only included GWAS summary statistics from European population, the generalizability of the results could be limited due to the ethnic differences. Third, several limitations of the LCV method exist [20]. For instance, the LCV method includes only a single intermediary and can be confounded when there are multiple intermediaries. In addition, while the LCV analysis employed genomic information, the statistical power of the original GWAS results could still impact the GCP estimates so that the capacity of the LCV analysis to identify the causal associations could be limited. Fourth, even though high GCP (|GCP| > 0.6) exits for many phenotype pairs, it is uncertain whether many of those phenotype pairs reflect fully or partially genetically causal associations. Finally, as mentioned in the previous study [20], genetic causality must be interpreted with caution before designing disease interventions, because interventions may fail to mimic genetic perturbations.

## Conclusions

In summary, 133 distinct phenotypes were identified to show causal relationships with OA through LCV approach. These phenotypes mainly involved anthropometrics, socioeconomic determinants, cardiovascular and metabolic disorders, soft tissue and musculoskeletal disorders, digestive/respiratory diseases, psychiatric conditions, pharmacological interactions. More studies are needed to validate and illustrate the detailed mechanism of those causal associations.

### Electronic supplementary material

Below is the link to the electronic supplementary material.

Supplementary Material 1

Supplementary Material 2

Supplementary Material 3

Supplementary Material 4

Supplementary Material 5

Supplementary Material 6

Supplementary Material 7

Supplementary Material 8

## Data Availability

All data generated or analyzed during this study are included in this published article and its supplementary information files.

## Abbreviations

OA: Osteoarthritis
MR: Mendelian randomization
GWAS: Genome-wide association studies
CTG-VL: Complex Traits Genomics Virtual Lab
LCV: Latent causal variable
GCP: Genetic causality proportion

## References

[1] Jaimie DS, Garland T, Culbreth., Lydia M, Haile (2023). Global, regional, and national burden of osteoarthritis, 1990–2020 and projections to 2050: a systematic analysis for the global burden of Disease Study 2021. Lancet Rheumatol.

[2] Allen KD, Thoma LM, Golightly YM (2022). Epidemiology of osteoarthritis. Osteoarthritis Cartilage.

[3] Macêdo MB, Santos V, Pereira RMR (2022). Association between osteoarthritis and atherosclerosis: a systematic review and meta-analysis. Exp Gerontol.

[4] Xie Y, Zhou W, Zhong Z (2021). Metabolic syndrome, hypertension, and hyperglycemia were positively associated with knee osteoarthritis, while dyslipidemia showed no association with knee osteoarthritis. Clin Rheumatol.

[5] Mohajer B, Kwee RM, Guermazi A (2021). Metabolic syndrome and Osteoarthritis Distribution in the hand joints: a propensity score matching analysis from the Osteoarthritis Initiative. J Rheumatol.

[6] Fonseca-Rodrigues D, Rodrigues A, Martins T (2021). Correlation between pain severity and levels of anxiety and depression in osteoarthritis patients: a systematic review and meta-analysis. Rheumatology (Oxford).

[7] Kamps A, Runhaar J, de Ridder MAJ (2023). Comorbidity in incident osteoarthritis cases and matched controls using electronic health record data. Arthritis Res Ther.

[8] Zengini E, Hatzikotoulas K, Tachmazidou I (2018). Genome-wide analyses using UK Biobank data provide insights into the genetic architecture of osteoarthritis. Nat Genet.

[9] Sun Y, Li Y, Yu T (2023). Causal associations of anthropometric measurements with osteoarthritis: a mendelian randomization study. PLoS ONE.

[10] Lyu L, Cai Y, Xiao M, et al. Causal relationships of General and Abdominal Adiposity on Osteoarthritis: a two-sample mendelian randomization study. J Clin Med. 2022;12(1). 10.3390/jcm12010320. 10.3390/jcm12010320 PMC9820884 36615120

[11] Yin M, Xu W, Pang J (2023). Causal relationship between osteoarthritis with atrial fibrillation and coronary atherosclerosis: a bidirectional mendelian randomization study of European ancestry. Front Cardiovasc Med.

[12] Wang Z, Kang C, Xu P (2022). Osteoarthritis and cardiovascular disease: a mendelian randomization study. Front Cardiovasc Med.

[13] Gill D, Karhunen V, Malik R (2021). Cardiometabolic traits mediating the effect of education on osteoarthritis risk: a mendelian randomization study. Osteoarthritis Cartilage.

[14] Tachmazidou I, Hatzikotoulas K, Southam L (2019). Identification of new therapeutic targets for osteoarthritis through genome-wide analyses of UK Biobank data. Nat Genet.

[15] Boer CG, Hatzikotoulas K, Southam L (2021). Deciphering osteoarthritis genetics across 826,690 individuals from 9 populations. Cell.

[16] Gabriel Cuéllar-Partida, Mischa Lundberg, Pik Fang Kho et al: Complex-Traits Genetics Virtual Lab: A community-driven web platform for post-GWAS analyses. bioRxiv et al. 2019:518027. 10.1101/518027.

[17] Haworth S, Kho PF, Holgerson PL (2021). Assessment and visualization of phenome-wide causal relationships using genetic data: an application to dental caries and periodontitis. Eur J Hum Genet.

[18] D’Antona S, Pathak GA, Koller D (2023). Phenome-wide genetic-correlation analysis and genetically informed causal inference of amyotrophic lateral sclerosis. Hum Genet.

[19] García-Marín LM, Campos AI, Kho PF (2021). Phenome-wide screening of GWAS data reveals the complex causal architecture of obesity. Hum Genet.

[20] O’Connor LJ, Price AL (2018). Distinguishing genetic correlation from causation across 52 diseases and complex traits. Nat Genet.

[21] Bulik-Sullivan BK, Loh PR, Finucane HK (2015). LD score regression distinguishes confounding from polygenicity in genome-wide association studies. Nat Genet.

[22] Karlsson T, Hadizadeh F, Rask-Andersen M (2023). Body Mass Index and the risk of Rheumatic Disease: Linear and nonlinear mendelian randomization analyses. Arthritis Rheumatol.

[23] Zhang L, Zhang W, Wu X (2023). A sex- and site-specific relationship between body mass index and osteoarthritis: evidence from observational and genetic analyses. Osteoarthritis Cartilage.

[24] Ni J, Wang P, Yin KJ (2022). Does smoking protect against developing osteoarthritis? Evidence from a genetically informed perspective. Semin Arthritis Rheum.

[25] He Y, Zheng C, He MH (2021). The causal relationship between body Mass Index and the risk of Osteoarthritis. Int J Gen Med.

[26] Yuan J, Wang D, Zhang Y (2023). Genetically predicted obesity and risk of hip osteoarthritis. Eat Weight Disord.

[27] Jiang L, Jiang Y, Wang A (2022). The causal association between bone mineral density and risk of osteoarthritis: a mendelian randomization study. Front Endocrinol (Lausanne).

[28] Hartley A, Sanderson E, Granell R (2022). Using multivariable mendelian randomization to estimate the causal effect of bone mineral density on osteoarthritis risk, independently of body mass index. Int J Epidemiol.

[29] Funck-Brentano T, Nethander M, Movérare-Skrtic S (2019). Causal factors for knee, hip, and Hand Osteoarthritis: a mendelian randomization study in the UK Biobank. Arthritis Rheumatol.

[30] Chen L, Jia C, Yang H (2023). Causal effect of higher glycated hemoglobin (HbA1c) levels on knee osteoarthritis risk: a mendelian randomization study. Rheumatol Ther.

[31] Xing X, Wang Y, Pan F (2023). Osteoarthritis and risk of type 2 diabetes: a two-sample mendelian randomization analysis. J Diabetes.

[32] Cui Z, Feng H, He B (2020). Type 2 diabetes and glycemic traits are not causal factors of Osteoarthritis: a two-sample mendelian randomization analysis. Front Genet.

[33] Arruda AL, Hartley A, Katsoula G (2023). Genetic underpinning of the comorbidity between type 2 diabetes and osteoarthritis. Am J Hum Genet.

[34] Chen H, Wu J, Wang M, et al. Impact of exposure to ambient fine Particulate Matter Pollution on adults with knee osteoarthritis. Int J Environ Res Public Health. 2021;18(18). 10.3390/ijerph18189644. 10.3390/ijerph18189644 PMC8466353 34574569

[35] Chau TT, Wang KY (2020). An association between air pollution and daily most frequently visits of eighteen outpatient diseases in an industrial city. Sci Rep.

[36] Peng KT, Liu JF, Chiang YC (2019). Particulate matter exposure aggravates osteoarthritis severity. Clin Sci (Lond).

[37] Liu JF, Chi MC, Lin CY (2021). PM2.5 facilitates IL-6 production in human osteoarthritis synovial fibroblasts via ASK1 activation. J Cell Physiol.

[38] Wang X, Perry TA, Arden N (2020). Occupational risk in knee osteoarthritis: a systematic review and Meta-analysis of Observational studies. Arthritis Care Res (Hoboken).

[39] Wang S, Liu Y, Wu K (2023). Osteoarthritis and risk of cardiovascular diseases: a mendelian randomization study. Injury.

[40] Steppuhn H, Langen U, Keil T (2014). Chronic disease co-morbidity of asthma and unscheduled asthma care among adults: results of the national telephone health interview survey German Health Update (GEDA) 2009 and 2010. Prim Care Respir J.

[41] Koo HK, Song P, Lee JH (2021). Novel association between asthma and osteoarthritis: a nationwide health and nutrition examination survey. BMC Pulm Med.

[42] Nie Y, Liu H, Wang J (2023). Systemic evaluation of the relationship between asthma and osteoarthritis: evidence from a meta-analysis and mendelian randomization study. Digit Health.

[43] Yan J, Zhang Y, Zhang X (2023). Increased risk of rheumatoid arthritis in patients with asthma: a genetic Association Study using two-sample mendelian randomization analysis. Arthritis Care Res (Hoboken).

